# Glial metabolic alterations during glaucoma pathogenesis

**DOI:** 10.3389/fopht.2023.1290465

**Published:** 2023-11-28

**Authors:** Anne Rombaut, Rune Brautaset, Pete A. Williams, James R. Tribble

**Affiliations:** Department of Clinical Neuroscience, Division of Eye and Vision, St. Erik Eye Hospital, Karolinska Institutet, Stockholm, Sweden

**Keywords:** glaucoma, metabolism, glia, microglia, astrocytes, Müller cells

## Abstract

Glaucoma is the leading cause of irreversible blindness. Current treatment options are limited and often only slow disease progression. Metabolic dysfunction has recently been recognized as a key early and persistent mechanism in glaucoma pathophysiology. Several intrinsic metabolic dysfunctions have been identified and treated in retinal ganglion cells to provide neuroprotection. Growing pre-clinical and clinical evidence has confirmed that metabolic alterations in glaucoma are widespread, occurring across visual system tissues, in ocular fluids, in blood/serum, and at the level of genomic and mitochondrial DNA. This suggests that metabolic dysfunction is not constrained to retinal ganglion cells and that metabolic alterations extrinsic to retinal ganglion cells may contribute to their metabolic compromise. Retinal ganglion cells are reliant on glial metabolic support under normal physiological conditions, but the implications of metabolic dysfunction in glia are underexplored. We highlight emerging evidence that has demonstrated metabolic alterations occurring within glia in glaucoma, and how this may affect neuro-glial metabolic coupling and the metabolic vulnerability of retinal ganglion cells. In other neurodegenerative diseases which share features with glaucoma, several other glial metabolic alterations have been identified, suggesting that similar mechanisms and therapeutic targets may exist in glaucoma.

## Graphical Abstract

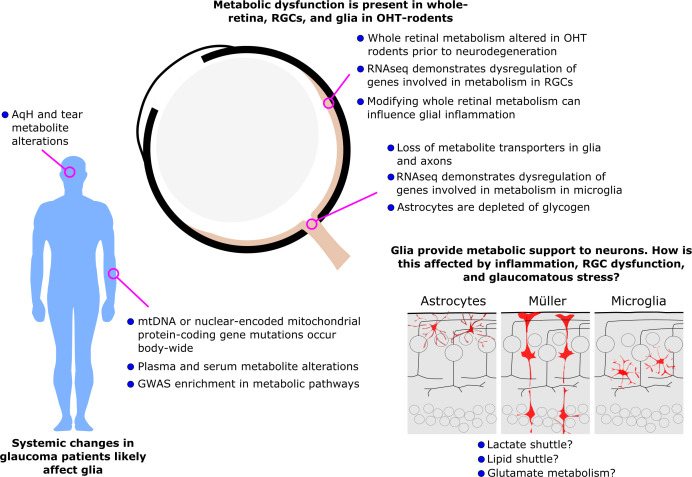

## 1 Introduction

Glaucoma is the most common cause of irreversible blindness worldwide, affecting at least 80 million diagnosed patients and approximately the same number again undiagnosed. The defining feature of glaucoma is progressive dysfunction and degeneration of retinal ganglion cells and their axons which make up the optic nerve. Many factors influence this progressive neurodegeneration including age, genetics, and high intraocular pressure (IOP) as well-established risk factors (1). Current treatments only target IOP, but many patients continue to lose vision in spite of IOP lowering, or fail to achieve IOP lowering (2). Hence, there is a pressing need for treatments that target the neurodegenerative process of glaucoma. Treatments targeting common neurodegenerative features could also more easily translate to other neurodegenerative diseases. Recently, metabolic dysfunction has been uncovered as a major feature of glaucoma pathology linking all three of these risk factors (3). There is increasing evidence of metabolic insufficiency either as an age-related loss of metabolic fitness, as a (genetic) predisposition or as a direct influence of ocular hypertension (high IOP). Mounting evidence for metabolic changes in glaucoma has now been identified across multiple animal models of glaucoma and in human glaucoma patients (3). Whilst the initial focus of these findings has been on retinal ganglion cells, much evidence has come from whole visual tissue, ocular fluids, blood, or DNA suggesting that metabolic dysfunction is likely not limited to retinal ganglion cells, but may also occur in supporting cells of the visual system. Glial cells perform vital homeostatic roles in supporting retinal ganglion cells, and while we know much about their importance in shaping the metabolic pathways of the retina under normal physiological conditions, we know little about how glial metabolism may be altered in glaucoma. This review summarizes the current knowledge on metabolic alterations in glia in glaucoma. For a detailed oversight of retinal metabolism, we direct the reader to a recent comprehensive review (4). The findings presented here predominantly come from rodent models which mostly reflect human primary open-angle glaucoma (POAG), although they have features of other subtypes. For animal data, the term ‘glaucoma’ is used to refer to experimental glaucoma. Human data, which supports a host of metabolic changes across the body, predominantly comes from POAG, but with some evidence from other subtypes. Where specific experiments are described, the subtype of glaucoma is noted, but when discussing implications, the term ‘glaucoma’ is used, and is predominantly used synonymously with POAG.

## 2 Metabolic dysfunction in glaucoma

### 2.1 Metabolic changes to retinal ganglion cells in glaucoma

Retinal ganglion cells are especially vulnerable to metabolic disturbances. To enable translucency of the retina, retinal ganglion cell axons have long unmyelinated regions and as such must propagate action potentials without saltatory conduction for a significant portion of their length. Retinal ganglion cells are not myelinated until their exit from the optic nerve head (ONH); a region termed the myeline transition zone (MTZ). This is a region of significant stress and susceptibility for retinal ganglion cells since their axons make a sharp turn to enter the ONH, where they are exposed to mechanical stress from ocular torsion and IOP. To maintain the energy demands from their lack of saltatory conduction and high activity, retinal ganglion cells require a high density of mitochondria, which is most dense at the MTZ. Like most neurons, the main metabolic pathways used by retinal ganglion cells to produce adenosine triphosphate (ATP) are glycolysis and oxidative phosphorylation (4). Glycolysis, which breaks down glucose to pyruvate, yields fewer mols of ATP per mol of glucose than oxidative phosphorylation but is a much more rapid process than the more tedious breakdown of pyruvate through oxidative phosphorylation (5). In executing these processes, retinal ganglion cells must sustain a delicate metabolic balance.

Retinal ganglion cell metabolism is dependent on the external availability of nicotinamide adenine dinucleotide (NAD), ATP, and other metabolites. NAD is an important redox cofactor involved in numerous key cellular processes. An age- and IOP-dependent decline in retinal NAD was first identified in DBA/2J mice as a feature of early disease (6). In this model, recessive mutations in the *Gpnmb* and *Tyrp1* genes result in iris pigment dispersion which block the trabecular meshwork causing an elevated IOP. The onset of this IOP increase is age-related but varies between individuals and colonies. This results in a variable onset of neurodegeneration, with typical moderate to severe neurodegeneration evident at approximately 12 months of age. (7). In the DBA/2J, downregulation of key NAD-producing enzymes (particularly *Nmnat2*) in retinal ganglion cells occurs prior to detectable neurodegeneration (6, 8). Decreased NAD in the retina and optic nerve has now also been identified in a rat model of ocular hypertension (9). In this model, paramagnetic beads are injected into the anterior chamber of the eye directed to the iridocorneal angle with an external magnet. This obstructs the trabecular meshwork and elevates IOP. IOP elevation is acute and can be induced independent of age. This results in moderate-to severe neurodegeneration within a short timeframe, typically 2-3 weeks. Supporting these findings in rodent models of glaucoma, in human tissue, reduced NMNAT2 labelling has been demonstrated in enucleated eyes from glaucoma patients (10). Mitochondrial dysfunction and degeneration have been identified in animal models of glaucoma and human tissue. Within the retina, a reduced volume of the mitochondrial crista was demonstrated by electron microscopy, occurring during ocular hypertension but prior to detectable neurodegeneration in the DBA/2J mouse (6). This was accompanied by widespread dysregulation of RNA transcripts encoding mitochondrial proteins and multiple metabolic pathways in retinal ganglion cells (6). It has yet to be determined whether this is a response to, or a driver of, metabolic dysfunction in retinal ganglion cells. Mitochondrial motility is also reduced in glaucoma, with disruption of mitochondrial transport at the ONH coinciding with high IOP. These data support the hypothesis of a block on mitochondrial transport at the ONH (11–15). Analysis of post-mortem retinas from human glaucoma patients has suggested that this likely also occurs in humans. Mitochondria were smaller and their distribution through retinal ganglion cell dendrites was more fragmented; this occurred in areas of the human glaucomatous retina with limited cell and visual function loss suggesting that mitochondrial dysfunction may precede gross neurodegeneration in humans with glaucoma (16).

### 2.2 Metabolic dysfunction in glaucoma is not constrained to retinal ganglion cells

Whilst RNA-sequencing of retinal ganglion cells has revealed a disruption to myriad metabolic transcripts, genetic analyses in humans have overwhelmingly relied on DNA sequencing. A wide range of mutations in either mtDNA or nuclear-encoded mitochondrial protein-coding genes has been identified in both POAG and normotensive glaucoma (NTG) patients (17–26). Additionally, an increased mtDNA content was shown in POAG patients (18). These DNA mutations will be present in all nucleated cells of the body, yet retinal ganglion cells are likely particularly vulnerable to these as is exemplified by mitochondrial diseases which predominantly only present with a visual phenotype (e.g. Leber hereditary optic neuropathy, autosomal dominant optic atrophy). However, while other ocular and retinal cells may be less reliant on mitochondrial and oxidative phosphorylation metabolism, there are some notable effects. Systemically, impaired activity of the mitochondrial Complex I has been identified in lymphoblasts of POAG patients, leading to reduced oxidative phosphorylation and less ATP production (27, 28). In Tenon’s ocular fibroblasts from glaucoma patients, impaired mitochondrial metabolism has been detected, resulting in a higher basal stress level (29). It is likely that mitochondrial mutations also affect glial cells of the retina and optic nerve (see section 4: “Glial metabolic changes in glaucoma”). Supporting this concept of a systemic metabolic susceptibility in glaucoma, several blood metabolites have been found to be altered in glaucoma patients compared to controls including citrate (30), nicotinamide (16), arginine (31, 32), glycine (33, 34), amongst many others (35).

Direct evidence of dysfunctional or disrupted metabolism within the retina has recently been identified in animal models of glaucoma. Metabolomics analysis of the whole retina in the DBA/2J has identified a decrease in pyruvate in the retina with increasing IOP. In the same mouse model, a decreased glycolytic capacity was demonstrated in the optic nerve (36). Accordingly, a decrease in retinal pyruvate level together with gene expression disruption of pyruvate and glycolysis metabolism and transport was identified by RNA sequencing in DBA/2J mice at an early stage in glaucoma development, and long-term oral administration of pyruvate has been shown to be neuroprotective in both rat and mouse models of glaucoma (36). In the DBA/2J, retinal ganglion cell axons were shown to have a decreased capacity to upregulate glycolysis when exposed to a higher metabolic demand (37). Similarly, in an inducible rat bead model of glaucoma, metabolic profiles were demonstrated to be dysregulated in the whole retina, optic nerve and superior colliculus, occurring at a timepoint when IOP was high but before detectable retinal ganglion cell degeneration (9), supporting a metabolic dysfunction across the whole visual system. Importantly, although some findings match specific gene changes in retinal ganglion cells, they reflect tissue metabolism as a whole. Given that glia, collectively, represent a significant proportion of cells in the retina and especially in the optic nerve, it is highly likely that some of these changes are derived from changes to glial metabolism or metabolic coupling of retinal ganglion cells and glia.

Metabolomics analyses of whole glaucomatous retina in animal models have identified potential changes to several metabolic pathways including alanine, aspartate and glutamate metabolism, D-glutamine and D-glutamate metabolism, and arginine biosynthesis (9, 36). Many of these metabolites and pathways have also been independently identified in human fluids including plasma, aqueous humor, and tears. According to Wang et al. (35) who reviewed metabolomics research in POAG, arginine (38, 39), and glycine (39) levels have been found to be altered in aqueous humour, while arginine is altered in tears. Pathway analysis of aqueous humour metabolites supports significant changes to arginine and proline metabolism, and taurine and hypotaurine metabolism. Similarly, for plasma metabolites, pathways analysis supports an alteration in arginine and proline metabolism accompanied by beta-alanine and sphingolipid metabolism changes in POAG. For serum metabolites glutathione, glyoxylate, dicarboxylate and biotin metabolism were altered (35). Differences in serum and plasma metabolite changes may reflect metabolic changes in blood cells. GWAS analysis of genetic glaucoma variants shows that the butanoate metabolism pathway was found to be significantly enriched in NTG, POAG, and high-tension glaucoma (40). Explanations for the importance of these altered metabolites in glaucoma pathology vary, and for many of these a logical clarification still needs to be found. These data suggest that amino acid metabolism likely plays an important role in glaucoma.

Supporting the widespread dysregulation of multiple metabolic pathways, human genetic analysis has also identified the potential for changes to mitochondrial lipid metabolism in both POAG and NTG. POAG was significantly linked to dysregulated fatty acid elongation and synthesis and the degradation of ketone bodies (40). Other studies have identified an adverse correlation between high levels of diglycerides and triglycerides and glaucoma (41) and the association of high total cholesterol levels and low HDL levels with glaucoma (42). When identifying the lipidomic profile of aqueous humour in open-angle glaucoma (OAG), Cabrerizo et al. (43) reported a higher level of most diacylglycerophosphocholines and 1-ether, 2-acylglycerophosphocholines, 14 sphingomyelins, and 5 cholesteryl esters in OAG patients compared to controls. These changes in lipid synthesis may signify a metabolic reaction to oxidative stress, or the utilization of lipids as an alternative energy source under the downregulation of oxidative phosphorylation, indicating an important role for lipids in glaucoma pathology. Supporting this, Williams et al. (6) showed increasing lipid droplet formation in DBA/2J mice with disease severity, which could be prevented by nicotinamide supplementation (which prevents metabolic and mitochondrial dysfunction).

Taken together, these lines of evidence support the potential for widespread metabolic dysfunction in glaucoma occurring systemically, and directly in the eye from IOP-related stress. Emerging evidence has now identified that glial, as well as retinal ganglion cells, exhibit metabolic changes in glaucoma. These likely reflect both a response to the metabolic dysfunction of retinal ganglion cells and direct pathological changes in glia in response to glaucomatous insults.

## 3 Retinal glia physiology and metabolism

Glia are fundamental to retinal function. The retina consists of three glial cell types: microglia, astrocytes, and Müller cells. Microglia are the resident macrophages of the retina, specialized in surveillance across all retinal layers during homeostasis, and in phagocytosis and peripheral immune recruitment when an irregularity is detected. Apart from immune signalling, microglia also have vital homeostatic functions such as the regulation of synapse formation and function in the retina. Astrocytes are cells with a more supportive function for the retinal ganglion cells. Astrocytes populate the nerve fibre layer and ganglion cell layer in the retina, and the ONH, where they envelop blood vessels. Much like their function in the brain, through tight junctions to blood vessels, they maintain the blood-retina barrier and immune privilege of the retina, regulate vascular tone, and support retinal ganglion cell axons metabolically through the transport of metabolites from the blood. Müller cells are specialized glia specific to the retina. In contrast to astrocytes, they span the entirety of the retinal layers and maintain the inner and outer limiting membrane of the retina. Here, Müller cells provide essential osmotic and (similarly to astrocytes) metabolic support to retinal ganglion cells (44). In the brain, glial cells and neurons exist in a roughly 1:1 ratio (although this varies by region), whereas glial cells are less abundant in the retina (3). This reflects another potential vulnerability of retinal ganglion cells to metabolic disruption. In neurons, the preferred energy source is glucose since this is a small molecule that can cross the different tight junction barriers bordering the central nervous system (45). The metabolic support of retinal ganglion cells mainly derives from astrocytes and Müller cells. This support is termed ‘metabolic coupling’ of glia and retinal ganglion cells and comprises different processes. For a detailed overview of the coupling between glia and neurons, we refer to Vecino et al. (46).

### 3.1 Glycogen storage

Astrocytes and Müller cells store glycogen (47). Glycogen can be produced from glucose and serves as a glucose-storage-molecule, that can be broken down into separate glucose units again when needed. In the eye, Müller cells have been found to produce more glycogen when extracellular insulin or K^+^ levels increase (48). This is consistent with the proposed role for glycogen to serve as an emergency reservoir for bioenergetic resources in the retina, where glycogen is synthesised in abundance of glucose and redistributed when retinal ganglion cells require extra energy *e.g.*, during hypoglycaemia or periods of high activity. This has also been confirmed for the astrocyte glycogen reserve in the brain (49). Gap junction connexin 43 (Cx43)-mediated astrocyte metabolic networks have even been found to serve the redistribution of glucose and other astrocyte-derived metabolites between optic nerves in mice (50).

### 3.2 The lactate shuttle

The transfer of lactate, a reduced form of pyruvate, from glia to neurons is termed the ‘lactate shuttle’. In many instances in the central nervous system, across different model organisms, astrocytes provide neurons with lactate as energetic support (51–53). Since both astrocytes and Müller cells express different monocarboxylate receptors, concentration-dependent (54) transport of lactate and pyruvate through both the blood-brain barrier and inner and outer blood-retina barrier is possible (55). However, it has been demonstrated that Müller cells predominantly account for lactate transfer in the retina. Müller cells predominantly produce ATP through ‘aerobic glycolysis’ or the ‘Warburg effect’: the process where glucose is preferably metabolized through glycolysis even though oxygen is present, sparing oxygen for the retinal neurons (56). However, Müller cells also express the necessary proteins for the TCA cycle. A minor proportion of the ATP in Müller cells is thus synthesized through oxidative phosphorylation (57). In a study conducted by Tsacopoulos et al. in the bumblebee retina, it was demonstrated that photoreceptor function in retinal slices could be maintained for several hours in a glucose-free environment, exclusively by retinal glia (58), suggesting the transfer of an energy substrate between glia and photoreceptors. Indeed, a few years later the same research group established in the guinea pig retina a net transfer of glycolytic lactate from Müller cells to photoreceptors fuelling oxidative phosphorylation (59) although this has yet to be established in species with vascular retinas. Lactate itself can also be used by Müller cells as an energy substrate since these cells preferably use glycolysis to produce ATP. The external administration of lactate enhances their function and survival (60), which may create a buffer for lactate overproduction in case of metabolic stress. Astrocytes also use a lactate-pyruvate shuttle in the retina since they have been shown to transfer lactate to retinal ganglion cells in the inner vascularized retina in a similar way as has been observed in the brain (61). Even though neurons demonstrate a preference for glucose over lactate as primary energy substrate (45, 62), the lactate shuttle could represent an important mode of protection of retinal ganglion cells against metabolic dysfunction (63). Lactate or pyruvate protects retinal ganglion cells from metabolic stress and injury *in vivo* (36, 64), and supplementing pyruvate in diet provides strong neuroprotection in glaucoma models (36).

### 3.3 The lipid shuttle

Glial cells have also been proposed to serve retinal ganglion cells by transferring lipids, termed the ‘lipid shuttle’. Here, astrocytes and Müller cells transfer lipids to retinal ganglion cells for synapse formation and axon maintenance, and photoreceptors for maintaining their highly membranous outer segments (65). Even though little is known about the exact mechanism, Müller cells and astrocytes have been found to synthesize high-density lipoprotein-like particles containing ApoE and ApoJ that were suggested to be secreted in the vitreous (66). Since retinal ganglion cells express microsomal triglyceride transfer proteins and photoreceptors can internalize lipid particles through the retinal pigment epithelium (67), they can internalize these lipoprotein-like particles.

### 3.4 Glutamate

The main neurotransmitter released by presynaptic neurons in the vertical pathways through the retina is glutamate (68). Glutamate is received by postsynaptic retinal ganglion cells and plays a role in the control of neuronal excitability. However, the accumulation of glutamate in the extracellular space is toxic to retinal ganglion cells. To prevent this, Müller cells express the protein EAAT1 (excitatory amino acid transporter 1), which allows them to rapidly take up glutamate (69). Once internalized, there are two possibilities for conversion of glutamate. In the first one, Müller cells convert glutamate to glutamine (70) to be taken up again by the presynaptic neurons for reconversion to glutamate (44). In the second possibility, glutamate is converted to α-ketoglutarate and feeds into the TCA cycle (71) as a key precursor for TCA cycle metabolites.

## 4 Glial metabolic changes in glaucoma

Inflammation in glaucoma is now well-established in animal models and humans. Microglia populations shift towards pro-inflammatory phenotypes which actively damage retinal ganglion cells. Astrocytes and Müller cells undergo reactive gliosis, and peripheral leukocytes infiltrate from the blood. This pro-inflammatory shift in the glia occurs both as a result of retinal ganglion cell neurodegenerative signalling and from direct IOP-related stress to the glia. The mechanisms and consequences of inflammation in glaucoma are well documented and will not be a focus of this review. For more detailed information about the influence of microglial (72–75), astrocyte (76), Müller cells (77), complement (78, 79) and oxidative stress (78), immune signalling or inflammation in general (80, 81) on glaucoma pathology, we direct the reader to other reviews. While inflammatory signalling mechanisms are well understood, we know very little about what metabolic changes occur because of, or even preceding, these inflammatory reactions. How this may contribute to neurodegeneration in glaucoma remains to be explored. Given the metabolic reliance of retinal ganglion cells on glia as highlighted above, it is likely that the shift in glial phenotypes away from supportive roles and towards pro-inflammatory phenotypes will lessen their ability to support retinal ganglion cells.

It is becoming increasingly apparent that glia themselves are under increasing metabolic stress in early glaucoma. The energy sensor and regulator AMPK is activated in response to decreases in intracellular ATP. This has been identified not only in retinal ganglion cells (82), but also in glia as demonstrated by significantly increased immunolabeling for phosphorylated AMPK in astrocytes in the DBA/2J optic nerve occurring early in disease (83). This may be in response to IOP-induced stress on glia, rather than purely a response to retinal ganglion cell metabolic dysfunction. Supporting this, Pappenhagen et al. (84) demonstrated that chronic stretching of ONH astrocytes *in vitro* (24 h of 12% biaxial stretch at 1 Hz) resulted in a significantly different glycolytic and respiratory activity between control and stretched cells. They observed greater extracellular acidification signifying an increased use of glycolysis, and lower ATP-linked respiration. However, higher maximal respiration (maximal oxygen consumption rate under high energy demand) and spare capacity (the difference between maximal and basal respiration) were observed in stretched ONH astrocytes compared to unstretched control cells, indicating an increased metabolic flexibility in glial cells during elevated intraocular pressure. For both stretched and control cells, glutamine was found as the preferred energy substrate. This together with the increased use of glycolysis, suggests a shift in stretched ONH metabolism to support a higher demand for energy by both glia and retinal ganglion cells. Whilst retinal ganglion cells are particularly vulnerable to metabolic disturbance, glia likely maintain some resilience to this in early disease and remain able to buffer neuronal metabolism. Cooper et al. (50) provide evidence for this, demonstrating an increasing depletion of glycogen (stored in astrocytes) in the optic nerve with the duration of IOP elevation. Supporting this, Wender et al. (85) demonstrated that glycogen reserves of astrocytes in the retina slow down action potential failure in rat optic nerve during hypoglycaemia by the breakdown of glycogen into lactate and the transfer of this lactate as an energy resource to retinal ganglion cells. Cooper et al. (50) demonstrated that glycogen depletion was greater in the optic nerve belonging to the unoperated eye, contralateral to the ocular hypertensive eye. They identified that this was mediated through Cx43 gap junctions, which increased astrocyte coupling after unilateral IOP-induced stress. This expanded network resulted in increased AMPK activation and glycogen depletion in the contralateral optic nerve, demonstrating the ability of glia to attempt to buffer against metabolic depletion. However, this also subsequently increased the vulnerability of the donating optic nerve to further metabolic insult, arguably because of the lack of energy reserves (50). This coupling therefore also propagates IOP-related metabolic stress through the visual system. Knocking out Cx43 protected the contralateral optic nerve, but accelerated metabolic dysfunction, axon transport deficits, and loss of visual function in the ipsilateral optic nerve exposed to high IOP (50), demonstrating both the importance of glial metabolic support to retinal ganglion cells and the finite capacity of this support to overcome retinal ganglion cell metabolic depletion.

RNA-sequencing of microglia in the optic nerve head of DBA/2J mice at an early time point (when IOP is high, but there is no detectable optic nerve degeneration) demonstrates that microglia also undergo an early disruption to the regulation of their metabolism (86). Microglia in DBA/2J ONHs demonstrate an upregulation of mitochondrial transcripts with predicted changes to oxidative phosphorylation, and dysregulation of genes involved in glycolysis, gluconeogenesis, and lipid metabolism (86). This may be explained by an increase in *Hif1a* expression, a master regulator of glycolysis, which is known to change in response to (oxidative) stress and inflammation. Supporting this, in a microbead mouse model of ocular hypertension (which follows the same principle as for the rat model explained above), hypoxia and an increase in *Hif1a* labelling were detected in microglia in the optic nerve and ONH (as well as in astrocytes and Müller cells (87). This was accompanied by an increase in the labelling of glucose transporters GLUT1 and GLUT3 suggesting an increase in uptake of metabolites (87). Similarly, in the microglia RNA-sequencing performed by our group, we demonstrated an increase in *Slc16a1* (MCT1) expression (86). MCT1 is a bi-directional transporter of lactate, pyruvate, or ketone bodies, supporting either the increased metabolic demands and versatility of microglia in early glaucoma, or increased metabolic support to retinal ganglion cell axons in the ONH. In contrast, GLUT1 labelling is significantly reduced in astrocytes in the optic nerve in early disease in the DBA/2J, while GLUT3 labelling in axons remained unchanged (83). This suggests that certain metabolic deficits may arise in glia prior to retinal ganglion cells. MCT1, MCT2, and MCT4 were significantly decreased in the optic nerve of DBA/2J mice and donor human optic nerve (83). Knockdown of MCT2 in the absence of disease is sufficient to reduce respiratory capacity in the optic nerve while its over-expression protects retinal ganglion cells from neurodegeneration in multiple mouse models of glaucoma (88). These findings support a constraint on the movement of energy substrates between axons and glia in the ONH and the early breakdown of neuro-glial metabolic coupling.

To counteract the low substrate (glucose) availability and the downregulation of monocarboxylate transporters, Harun-Or-Rashid et al. fed DBA/2J mice a ketogenic diet. In this diet, the subject is restricted to a high amount of fat and a low amount of carbohydrates mimicking fasting conditions for the body, and results in the production of ketone bodies. As a consequence, the metabolism of the subject is altered towards increased fatty-acid oxidation and gluconeogenesis, while glycolysis is limited. This leads to the increased production of acetyl-CoA (from fatty acid oxidation) and an increased flow of oxaloacetate out of mitochondria as support for gluconeogenesis and the generation of ketone bodies. Ketone bodies and fatty acid oxidation provide an alternative energy source to glucose. DBA/2J mice on a ketogenic diet did not exhibit monocarboxylate transporter decline and retinal ganglion cells were protected. A robust antioxidant response accompanied these effects (83). In further research, the same group showed that a ketogenic diet resulted in lower microglial and astrocytic activation rates, production of anti-inflammatory factors such as IL-4, arginase-1 and hydroxycarboxylic acid receptor 1 in the DBA/2J retina and optic nerve. This was accompanied by low AMPK phosphorylation, also reducing NF-κB p65 signalling, TNF-α, IL-6, and NOS2 expression in DBA/2J retina and optic nerve (89). It is unknown whether this limit in glial inflammation is directly attributable to an increase in alternative energy substrates available to the glia themselves, or as a result of reduced intrinsic metabolic dysfunction and degeneration of retinal ganglion cells lessening the downstream stress on glia (or a combination of both). Brain microglia in aged mice upregulate oxidative phosphorylation and utilization of ketone energy sources, reflecting either a stress response or loss of transcriptional regulation (86), which suggest that glia are at least capable of directly using these alternative energy sources. In other contexts where metabolic support is provided to retinal ganglion cells in glaucoma, inflammation persists (*e.g. Wld^S^* providing increased NAD resulting in strong neuroprotection, (90) while in other cases it is reduced (*e.g.* pyruvate supplementation) (36). This discrepancy may reflect the differing accessibility, utility, and importance of these metabolites to retinal ganglion cells and glia.

Disruption to glial metabolism and metabolic pathways could have far-reaching consequences in not only their ability to support retinal ganglion cells but also in producing directly neurotoxic responses. As discussed above, mitochondrial dysfunction, particularly mitochondrial mutations, is likely to affect glia in glaucoma. Vohra et al. (91) demonstrated that disrupting mitochondrial activity in cultured MIO-M1 human Müller cells results in decreased basal ATP turnover and maximal respiration. Additionally, both glutamate uptake and mRNA expression of the glutamate transporter EAAT1 deceased. If this reproducibly occurs *in vivo*, then this would translate to a decrease in glutamate uptake from the intracellular space, contributing to enhanced ecotoxicity and retinal ganglion cell death. Supporting this possibility, the K+ channel Kir4.1 is disturbed in Müller cells in ocular hypertensive conditions (92), impairing the uptake of glutamate (93). Disturbance in mitochondrial metabolism may also have important consequences for oxidative stress. Oxidative stress has been detected in retinal glia in ocular hypertensive models of glaucoma. Jassim et al. (94) demonstrated that retinal levels of glutathione were significantly decreased after IOP elevation compared to control, and hypoxic Müller cells, microglia, astrocytes and retinal ganglion cells were present after 4 weeks of ocular hypertension but absent in normotensive control retinas (pimonidazole-positive, defined as less than 10 mmHg O_2_). Additionally, the mean intensity of dihydroethidium (indicative of superoxide levels) was significantly increased in the ganglion cell layer and ONH, and an increased LC3-II to LC1-I ratio suggested an increase in autophagic activity in hypertensive retinas compared to control. Taken together these data suggest that damaging high IOP can have a direct consequence, not only on retinal ganglion cell metabolism, but also on glia metabolism and stress. The consequences of glial metabolic change on their immune roles also remain to be explored. RNA-sequencing of microglia in the DBA/2J ONH, early in disease, demonstrated the dysregulation of several sensome, inflammatory, and phagocytic pathways concomitant with the dysregulation of metabolic pathways (86). Evidence from other neurodegenerative diseases suggests that the accumulation of mtDNA mutations itself may act as a trigger of microglial activation (95), supporting the possibility that metabolic changes in glia may alter glial inflammatory responses. There is strong evidence linking purinergic signalling with microglia activation in glaucoma, and while evidence from animal models supports the early increase in ATP release from numerous retinal cell types in response to direct IOP stress and inflammatory activation, the consequences of shifting metabolic preferences on these signalling pathways has yet to be explored. Increased levels of ATP in the aqueous humor of older adults and POAG patients (96) and in ocular tissues in experimental glaucoma and patients (97, 98), suggest that ATP release as a signalling molecule continues despite metabolic dysfunction. While direct evidence of glial metabolic change in glaucoma is currently limited, the likelihood of uncovering further changes is high given recent findings in other neurodegenerative diseases.

## 5 Lessons from other neurodegenerative diseases

Neurodegenerative diseases have many mechanisms in common including intrinsic neurodegenerative pathways, neuroinflammation, and metabolic dysfunction. Alzheimer’s disease (AD), Parkinson’s disease (PD), amyotrophic lateral sclerosis (ALS), multiple sclerosis (MS), Huntington’s disease (HD), and other less common neurodegenerative diseases often have long periods of pathology development and neurodegeneration before the disease is clinically detected. Patients diagnosed with glaucoma are more prone to develop another neurodegenerative disease, while glaucoma often develops in patients diagnosed with a neurodegenerative disease ((99) for AD and PD, (100) for MS). A glaucoma-like phenotype in the eye, resulting in the degeneration of the optic nerve is common in many neurodegenerative diseases (AD (101–104); PD (105, 106); ALS (107, 108); MS (103, 109)). This topic has been reviewed more extensively in (110). Metabolic dysfunction is now recognized as an important disease component across many neurodegenerative diseases, particularly the loss of NAD homeostasis, increased mitochondrial dysfunction, and a metabolic switch towards alternative energy sources. In these diseases, several direct metabolic changes in glia have been identified, and given the similarities of these diseases to glaucoma, it seems highly likely that many of these metabolic changes could also occur in glaucoma.

Ageing is a common risk factor for neurodegenerative disease. Increasing age leads to the senescence of glia in the central nervous system, affecting their inflammatory and homeostatic functions. Increasing evidence demonstrates that glial senescence causes intrinsic metabolic changes to glia which also affect their response to neurons and neurodegenerative disease. Lipid droplet accumulating microglia have been found in the hippocampus of ageing mice. Lipid droplet accumulating microglia have a reduced phagocytic capacity and release inflammatory mediators that promote age-related inflammation (111). Lipid deposits in aged microglia impair the cellular responses to ischemia (112), while ischemia induces microglial changes in lipid metabolism (113). Astrocyte-specific oxidative phosphorylation deficiency induces synaptic loss, neuroinflammation, demyelination, cognitive impairment, and metabolic and transcriptional signatures with significant overlap with mouse models of AD. This aberrant oxidative phosphorylation in astrocytes results in lipid droplet accumulation, suggesting a clear importance of lipid metabolism as a mechanism underlying AD pathology, where astrocytic mitochondrial dysfunction progressively induces neuroinflammation and neurodegeneration (114). Lipid accumulation in the retina has previously been identified in the DBA/2J (although this has yet to be localized to a specific cell type) suggesting that similar lipid-mediated metabolic defects could be present in retinal glia and could contribute to neuroinflammation in glaucoma.

Genetic variants of TREM2 are associated with AD, PS, and ALS (115, 116), which suggests a common mechanism for pathology development in multiple neurodegenerative diseases. TREM2 is a lipid-sensitive receptor expressed in microglia that mediates phagocytosis, particularly of myelin debris. Microglia deficient in TREM2 are still able to internalize myelin debris but are unable to remove the myelin cholesterol (117). One of the pathological characteristics of MS is microglia containing incompletely processed myelin. In mouse models of AD, pathological amyloid beta plaques are internalized in microglia in a TREM-2-dependent manner. Additionally, in AD mouse models, the expression levels of TREM2 have been shown to affect mitochondrial mass (118). *TREM2* variants associated with AD do not significantly contribute to POAG risk, while the apolipoprotein E ϵ4 (APOE4) allele is associated with reduced risk (119). APOE4 drives lipid metabolism dysregulation in astrocytes and microglia in AD (120), but reduces neurodegeneration in glaucoma by limiting microglial activation (121). These highlight potential differences in the metabolism and neuroinflammatory responses in the retina and optic nerve which may differ from the brain. Exploring whether, in glaucoma, microglia in the brain (e.g., in the superior colliculus or dorsolateral geniculate nucleus where retinal ganglion cell axons terminate) share responses and characteristics with those in glaucomatous retina or those in the AD brain would be a valuable series of experiments. Similarly, as the retina is implicated in AD, further experiments could explore differences between retina and brain effects within the same AD mouse or AD patient tissue.

The transition of immune cells towards a pro-inflammatory state requires the rapid expression of an alternate proteome (production of cytokines and chemokines, immune receptors, and phagocytic machinery). This requires the rapid mobilization of energy, for which immune cells will undergo a Warburg-like effect favouring glycolysis and the utilization of lipid energy sources. This likely undermines the homeostatic energy roles of glia. Hyperglycolysis and the induction of the key glycolytic enzyme hexokinase 2 is essential for the development of microglia-mediated neuroinflammation during hypoxia (122). In primary astrocyte cell cultures derived from the SOD1 mutant mouse model of ALS, defects in oxidative phosphorylation have been identified (123). In astrocytes derived from *C9orf72* ALS patients, a lower percentage of ATP is produced in mitochondria compared to controls, suggesting a higher rate of glycolysis (124). Supporting this, in spinal cord gliosomes derived from SOD1 mutant mice, an increased activity of lactate dehydrogenase was detected, while this was not seen in spinal cord synaptosomes (neurons) (125). The cell-type specific transcriptional profiling of mutant SOD1 mice shows early abnormalities in two nuclear receptors, the peroxisome proliferator-activated receptor and liver X receptor, which regulate mitochondrial biogenesis, oxidative metabolism, and mitochondrial fatty acid metabolism (126). Similarly, in the HdhQ(150/150) mouse model of HD, striatal astrocytes have altered NADH/NAD+ ratio, reduced complex II levels and activity, increased fatty acid oxidation, and increased reactive oxygen species (127). In mouse models of PD and PD-related Gaucher disease, mitochondrial structural and functional abnormalities were demonstrated in glial cells (128, 129). In iPSC-derived astrocytes harbouring mutations in presenilin, which leads to early onset AD (130), lactate metabolism is significantly altered. These suggest that not only do glia transition to alternative energy sources as part of immune responses, but that they may undergo similar mitochondrial and metabolic dysfunction as has been identified in neurons. The evidence of metabolic alterations in glial cells in neurodegenerative diseases is increasing and is thus not only a potential avenue for the discovery of new mechanisms of glaucomatous neurodegeneration but also a very attractive target for future therapies.

## 6 Conclusions

Metabolic alterations in glaucoma are widespread and varied. Alterations occur at the level of genomic and mitochondrial DNA, retinal cells, visual system tissues, ocular fluids, and blood/serum. Metabolic alterations and dysfunction in retinal glia is an emerging research topic and demonstrates potential mechanisms that may contribute to retinal ganglion cell metabolic dysfunction. Current evidence suggests not only that glia may share some metabolic perturbations with retinal ganglion cells, but that they may also represent shared neuroprotective targets for glaucoma treatment.

## Author contributions

AR: Writing – original draft, Writing – review & editing. RB: Supervision, Writing – review & editing. PW: Conceptualization, Funding acquisition, Resources, Supervision, Writing – review & editing. JT: Conceptualization, Funding acquisition, Supervision, Writing – original draft, Writing – review & editing.
